# Insights into the Species Diversity and Features of Fungi in the *Fusarium heterosporum* Species Complex

**DOI:** 10.3390/jof12060416

**Published:** 2026-06-08

**Authors:** Olga P. Gavrilova, Aleksandra S. Orina, Nadezhda N. Gogina, Tatiana Yu. Gagkaeva

**Affiliations:** 1 Laboratory of Mycology and Phytopathology, All-Russian Institute of Plant Protection, 196608 St. Petersburg, Russia; orina-alex@yandex.ru (A.S.O.); t.gagkaeva@yahoo.com (T.Y.G.); 2 Laboratory of Biochemical Analysis, All-Russian Scientific Research and Technological Institute of Poultry, 141311 Sergiev Posad, Russia; n.n.gogina@mail.ru

**Keywords:** phylogeny, morphology, mating type, sexual reproduction, growth rate, moniliformin

## Abstract

In this study, four *Fusarium* strains isolated from Poaceae plants infected by *Claviceps* spp. and one strain isolated from the stem of *Cirsium arvense* collected from two regions of Russia that are separated by a long distance were analyzed in detail. These fungi were accurately identified through a phylogenetic analysis of the fragments of translation elongation factor 1-α and RNA polymerase second largest subunit loci. Four of them belong to *F. heterosporum* species, and one strain, MFG 13060, together with the historical reference strain BBA 62226, forms a distinct lineage within the *F. heterosporum* species complex (FHSC). The morphological features of the anamorph structures of the fungi within the FHSC are presented. All the analyzed *F. heterosporum* strains are heterothallic and require a partner to mate. The fertile perithecia of *F. heterosporum* were obtained in a crossing experiment, and the teleomorph structures were characterized in detail. The screening of 19 mycotoxins typically produced by *Fusarium* fungi using high-performance liquid chromatography with tandem mass spectrometry revealed the ability of the strains to produce only moniliformin on an autoclaved rice substrate. A reassessment of the species diversity, distribution, and significance of fungi belonging to the FHSC is necessary to elucidate the unclear relationships between *F. heterosporum*, *Claviceps* fungi, and cereal plants.

## 1. Introduction

In accordance with modern *Fusarium* taxonomy, the *F. heterosporum* Nees & T. Nees species heads the *F. heterosporum* species complex (FHSC), which includes two other species. One is *F. graminum* Corda, and the other is the recently described *F. qiannanense* H. Zhang & Y.L. Jiang, strains of which were isolated in China from visually healthy roots of *Rosa roxburghii* [1] and from the asymptomatic sclerotium of *Claviceps purpurea* [2].

*Fusarium heterosporum* has been traditionally classified in the Section DISCOLOR Wollenw. (Section FUSARIUM in the taxonomic system of the Gerlach & Nirenberg [3]) in genus *Fusarium* and characterized on the basis of the morphological features of the asexual morph. In the research of Crous et al. [4], there are 19 taxonomic synonyms of *F. heterosporum*, including *F. lolii* (Wm.G. Sm.) Sacc. (1895); *F. heterosporum* var. *lolii* (Wm.G. Sm.) Wollenw. (1931); *F. heterosporum* f. *paspali* Ellis & Everh., in Ellis (1886); *F. heterosporum* var. *paspalicola* (Henn.) Wollenw. (1931); *F. congoense* Wollenw (1916); and *F. heterosporum* var. *congoense* (Wollenw.) Wollenw. (1931).

*Fusarium graminum* is recognized by Wollenweber & Reinking (1935) and Gerlach & Nirenberg (1982) and has been classified in the Section ROSEUM Wollenw. The synonyms of *F. graminum* [4] are *F. herbarum* var. *graminum* (Corda) Wollenw. (1930); *F. avenaceum* var. *graminum* (Corda) Raillo; and *F. corallinum* Sacc., Nuovo Giorn. (1876).

The similarities between these species include whitish, pale peach, flesh, yellowish to ochraceous base pigmentation; the absence of a distinct red pigment; and similar conidiogenesis, shape and size of conidia. Major differences include the formation of chlamydospores in *F. heterosporum* and their absence in *F. graminum* [3]. A karyotype study of *Fusarium* species revealed that the strains of *F. heterosporum* NRRL 20693 (CBS 720.79 = PD 79/878) and *F. graminum* NRRL 20692 (CBS 737.79 = BBA 62228) have seven core chromosomes, and *F. heterosporum* has an additional noncore chromosome [5]. According to the publications of Booth [6] and Ali et al. [7], there are no significant differences between *F. graminum* and *F. heterosporum*; thus, the name *F. heterosporum* is preferred since it has nomenclatural priority [8].

The taxonomic position of these species has been subject to debate, particularly with regard to their distinction from other closely related *Fusarium* species. Furthermore, data concerning the reproductive lifestyle and the occurrence of sexual reproduction in these species are conflicting; thus, it is necessary to clarify what genetic mating system is typical for these species [6,7].

The ecological role and requirements of fungi belonging to the FHSC are poorly understood and deserve to be studied more extensively. The range of plants infected by these fungi is generally limited to plants of the Poaceae family, including cultivated and wild cereals worldwide [9,10]. *Fusarium heterosporum* is also associated with ergot caused by *Claviceps* spp., since this fungus is often isolated from sclerotia [7,11,12,13,14,15,16]. Epitypus of *F. heterosporum* (CBS 391.68) was collected in Germany on the sclerotium of *Claviceps purpurea* on *Lolium perenne*, Aug. 1967, U.G. Schlösser [4]. The frequent isolation of *F. heterosporum* from grasses infected by *Claviceps* spp. suggests the presence of a biological system in which the relationships between these organisms deserve further investigation.

The aim of this study was to identify and characterize strains belonging to the *F. heterosporum* species complex isolated from Poaceae plants infected by ergot.

## 2. Materials and Methods

### 2.1. Fusarium Strains

**Figure 1:**
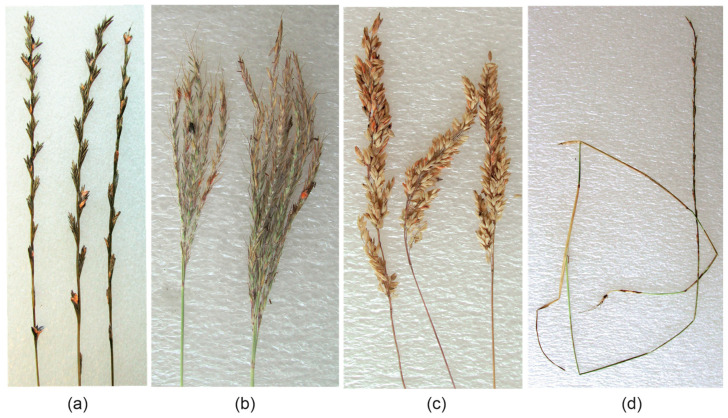
The plant sources of isolated *Fusarium* strains: (**a**). *Lolium* sp., LEP 65302; (**b**). *Cynodon dactylon* (L.) Pers., LEP 65303; (**c**). *Elytrigia* sp., LEP 65301; (**d**). *Poa* sp., LEP 65304.

Previously, four strains of *Fusarium* fungi were isolated from cereal plants. All the plants were infected by *Claviceps* spp., and visually noticeable orange sporulation of *Fusarium* fungi on the sclerotia and spikelets was detected (Figure 1). In addition, one strain was isolated from the stem of *Cirsium arvense* (L.) Scop. The strains were single-spored and phenotypically identified as representatives of the FHSC; after this, they were maintained in the fungal culture collection of the Laboratory of Mycology and Phytopathology of the All-Russian Institute of Plant Protection (St. Petersburg, Russia). The cereal plants from which *Fusarium* strains were isolated are stored in the Mycological Herbarium, with acronym LEP.

### 2.2. DNA Extraction, PCR, Sequencing and Phylogenetic Analysis

*Fusarium* strains were subsequently grown on potato dextrose agar (PDA) for 7 days. Genomic DNA was isolated from fungal mycelia (10–50 mg per strain) using the CTAB protocol [17]. The amplification of parts of the translation elongation factor 1-α (*tef*) and RNA polymerase second largest subunit (*rpb2*) genes was performed as described previously [18,19,20]. Amplicons were sequenced on an ABI Prism 3500 sequencer (Applied Biosystems, Hitachi, Japan) using the BigDye Terminator 3.1 cycle sequencing kit (Applied Biosystems, Foster City, CA, USA). The consensus sequences of each strain were obtained and manually edited using the Vector NTI Advance 10 program (Thermo Fisher Scientific, Carlsbad, CA, USA) and then deposited in the NCBI GenBank (Table 1). The Basic Local Alignment Search Tool (BLAST 2.17.0.) and FUSARIOID-ID database [21] were used to search for and select reference *Fusarium* strains. The sequences of representative strains from the NCBI GenBank database were included in the phylogenetic analysis (Table 2).

Sequence alignment was completed using MEGA X 10.1 [22]. Bayesian analysis was performed with MrBayes 3.2.1 on the Armadillo 1.1 platform [23] using 2,000,000 generations of Markov chain Monte Carlo, and trees were sampled every 1000 generations. The best-fitting substitution model was determined, and likelihood analysis was conducted with the IQ-TREE2 v.2.1.3 program [24]. For multilocus analysis, the TNe + I model was chosen. Nodal bootstrap support in maximum likelihood analysis was assessed on the basis of 2000 replicates.

### 2.3. Morphological Characterization

The strains were studied using a combination of morphological features commonly used to describe *Fusarium* fungi [8,25].

The morphology and growth of the colonies were determined by the cultivation of the strains on potato dextrose agar (PDA), synthetic nutrient agar (SNA), and carnation leaf agar (CLA). All media were prepared in-house, contained 1.5% agar and were maintained at approximately pH 6.0 [8,26]. The strains were initially grown on PDA for 7 days at 25 °C. The mycelial plugs of each strain (4 mm diameter) cut from the margins of the colony were individually placed surface downward on a medium in the center of each Petri dish. All the plates were incubated at 25 °C for 6–14 days either in the dark or under a 12 h light/dark cycle.

The shape, septation, and size of the conidia and the presence or absence of chlamydospores were observed for fungi grown on CLA and SNA. Microscope slides were prepared by mounting structures in a drop of water or taking photos of intact mycelia in situ on pieces of agar medium at 20–40–60× magnification. Whenever possible, a minimum of 30 measurements were made per structure under a light microscope at 60× or 100× magnification. Microscopic examination and imaging were performed with an Olympus BX53 microscope and an Olympus SZX16 stereomicroscope (Olympus, Tokyo, Japan). Images were captured with Jenoptik Gryphax PROKYON (Jenoptik AG, Jena, Germany) and edited with Adobe Photoshop CC 2018 (Adobe System Incorporated, San Jose, CA, USA).

### 2.4. Cultivation of Fungi at Different Temperatures

All the strains were previously grown on PDA for 7 days in the dark at 25 °C. Plates with pure PDA 85 mm in diameter were inoculated in the center with 4 mm plugs taken with a sterile cork-borer from the margin of an actively growing fungal culture. Each strain was cultured on PDA at 5, 10, 15, 20, 25, 30 and 35 °C in the dark for 6 days in an Innova 44R thermostat (Eppendorf, Hamburg, Germany).

The size of each colony was measured on day 6 in two perpendicular directions, and the average diameter of the colony minus the diameter of the inoculation plug was calculated. The growth rate of the strain was calculated as the ratio of the diameter of the fungal colony to the number of days of cultivation (mm/day).

### 2.5. Mating Type-Specific PCR and Crossing Experiment

The mating type of the fungal strains was determined by a PCR assay with the primer pairs fusALPHAfor/fusALPHArev and fusHMGfor/fusHMGrev according to the protocols and thermal conditions described by Kereni et al. [27]. The 200 bp and 260 bp sequences of the *MAT1-1* and *MAT1-2* idiomorphs, respectively, of the mating-type locus were amplified by these pairs.

Carrot agar (CA) was developed for fertility studies of *Fusarium* species [8], but we used a modified medium, since it has been proven to be well suited for producing the sexual stage of *Fusarium* fungi under laboratory conditions. Briefly, after washing, peeling and dicing, 200 g of fresh carrot was added to 200 mL of water and homogenized into a smoothie by a maker blender NB100DG Pro (Nutribullet, Ningbo, Zhejiang Province, China). Another 800 mL of water and 20 g of agar were added to the mixture, and the obtained medium was poured into vessels and then autoclaved for 30 min at 121 °C.

The stems of sweet clover (*Melilotus albus* Medik.), 2.5–5.0 mm in diameter, were collected during and after flowering; stripped of small branches, leaves, and flowers; and cut into 4–5 cm pieces. Fresh or frozen (−18 °C) stem pieces were rinsed under running water and sterilized with a 5% sodium hypochlorite solution for 5–10 min. Then, the pieces of stems were washed with sterile water and blotted with sterile filter paper, and 2–3 pieces were placed on the surface of the CA in Petri dishes.

The aerial mycelia of strains previously grown on PDA were transferred to CA with *Melilotus* stems (CMA), and 50 µL of Tween-60 (10%) water solution was added. For heterothallic fungi, the mycelia of each strain of the opposing mating types were added to one Petri dish. The fungal mycelium was mixed thoroughly with the stem pieces using tweezers and spread across the surface of the medium. The CMA medium promoted the abundant formation (up to 3 weeks) of fertile perithecia of fungi when they had this genetically determined capability. In the crossing experiments, all the combinations of the paired strains were tested for their ability to undergo sexual reproduction. All cross experiments were carried out under a photoperiod of 12 h UV light/12 h darkness cycles at 23–24 °C. Perithecia formation was monitored over the next 14–21 days. Some images of the perithecia were obtained by examining them in a drop of lactic acid when they turned red. Images were obtained as described above for the anamorphic stage of the fungus.

### 2.6. Mycotoxin Analysis

All the strains were tested for their ability to produce mycotoxins, which are commonly analyzed in *Fusarium* fungi. High-performance liquid chromatography with tandem mass spectrometry (HPLC–MS/MS) was used to detect 19 mycotoxins produced by *Fusarium* fungi, including trichothecenes (T-2 toxin, HT-2 toxin, T-2 triol, neosolaniol, diacetoxyscirpenol, deoxynivalenol (DON), 3-AcDON, 15-AcDON, DON-3-glucoside, nivalenol, and fusarenone-X), zearalenone, α-zearalenol and β-zearalenol, moniliformin (MON), beauvericin, and fumonisins B1, B2, and B3 (FUMs).

Flasks containing 20 g of polished rice and 12.5 mL of water were autoclaved (30 min at 1 atm). In every flask, the rice substrate was mixed with two agar plugs (5 mm diameter) taken from the cultures growing on PDA. All flasks were stored at 25 °C for three weeks in an Innova 44R thermostat (Eppendorf, Germany) and shaken every second day. Afterward, the samples were dried at 55 °C for one day and homogenized separately in sterilized grinding chambers of a batch mill (IKA; Königswinter, Germany).

Mycotoxin analysis was carried out according to the standard method [28]. The extraction of mycotoxins was carried out from 5 g of every flour obtained by adding 20 mL of extraction solvent (acetonitrile/water/acetic acid, 79:20:1, *v*/*v*/*v*) and mixing on a PSU-20 rotary shaker (Biosan, Riga, Latvia) for 90 min. The extracts were subsequently centrifuged for 2 min at 3000 rpm (Polycom CLn-16, Moscow, Russia). Each 500 μL extract without any purification was transferred to a glass vial, and 500 μL of a solution of acetonitrile:water:acetic acid (20:79:1 *v*/*v*/*v*) was added. Afterward, the vials were sealed and shaken for 30 s on a Vortex Genius3 (IKA, Königswinter, Germany).

Five microliters of the extract solution from each sample was collected using an Agilent autosampler (Agilent Technologies, Waldbronn, Germany) and separated into analytes using gradient elution on a Phenomenex Gemini (Torrance, CA, USA) C18 150 mm × 4.6 mm, 5 μm chromatographic column at 25 °C. Mycotoxins were detected on an AB SCIEX Triple Quad™ 5500 MS/MS system (Applied Biosystems, Framingham, MA, USA) equipped with a TurboV electrospray ionization source in positive and negative ionization modes. Analyst Software 1.6.2 and MultiQuant Software 3.0.2 were used to interpret the signals and calculate the quantitative content of the mycotoxins.

The mycotoxins were quantified by comparing peak areas with calibration curves obtained with standard solutions (Romer Labs Diagnostic GmbH, Tulln, Austria).

The limit of detection (LOD) and limit of quantification (LOQ) for moniliformin were 1.87 and 2.43 μg/kg, respectively.

### 2.7. Statistical Analysis

The data were analyzed using Microsoft Office Excel 2010 (Microsoft, Redmond, WA, USA) and Statistica 12.0 (StatSoft, Tulsa, OK, USA). The significance of differences between the mean values of groups was estimated via Tukey’s test (95% confidence level).

## 3. Results

### 3.1. Molecular Phylogeny

**Figure 2:**
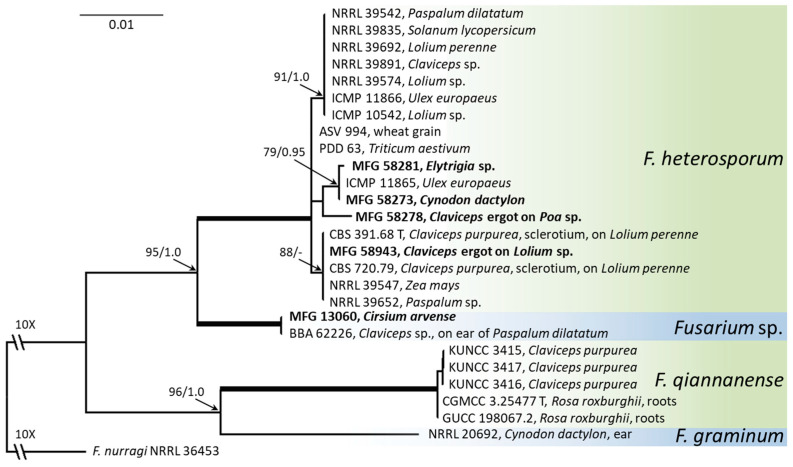
Maximum likelihood phylogenetic tree based on DNA sequence data from two loci (*tef*, *rpb2*) of *Fusarium* spp. ML bootstrap support values > 70%, followed by Bayesian posterior probability scores > 0.95 shown at nodes. Thickened lines indicate ML of 100 and BP of 1.0. Strains’ collection numbers and substrate are provided. Studied strains are in bold. T—type strain. Tree was rooted on sequences of *F. nurragi* strain NRRL 36453.

A multilocus analysis of the *tef* and *rpb2* sequences was used to infer the genetic relationship between *Fusarium* strains within the FHSC. The dataset included the combined sequences of the five analyzed strains as well as the sequences of the 23 reference *Fusarium* spp. strains and consisted of 1517 characters (614 bp from *tef* and 903 bp from *rpb2*), among which 1205 characters were conserved, and 264 were variable (17.4%); 119 characters were parsimony-informative (7.8%). The *Fusarium nurragi* (Summerell & Burgess) Benyon, Summerell & Burgess NRRL 36453 strain was used as an outgroup (Figure 2).

The four analyzed strains clustered together with the 14 reference *F. heterosporum* strains with high bootstrap support (ML/BP 100/1.0): one strain was included in a subclade with the *F. heterosporum* type strain CBS 391.68, whereas three strains were distributed into a sister subclade. One strain, MFG 13060, formed an unambiguous clade with the reference strain BBA 62226 (ML/BP 100/1.0). The obtained data support the existence of a distinct phylogenetic lineage within the FHSC.

### 3.2. Morphology Description

***Fusarium heterosporum* strains**

**Figure 3:**
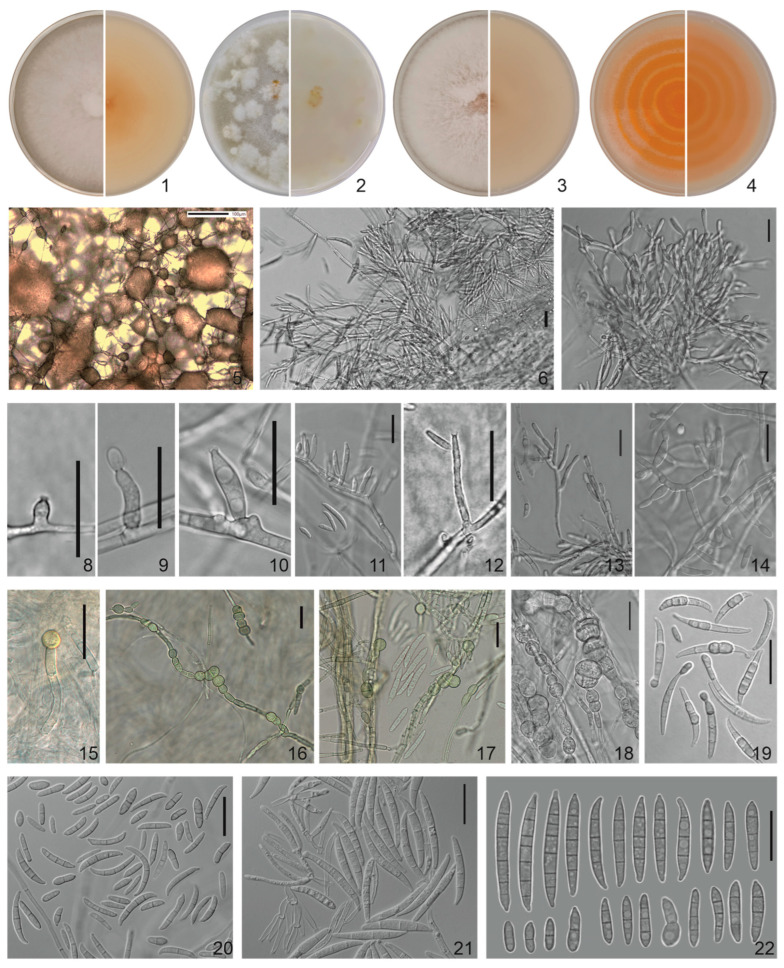
*Fusarium heterosporum*. Colony morphology on PDA of strains MFG 58943 in 1 week (**1**) and 3 weeks (**2**), in dark, and MFG 58281 in 1 week, in dark (**3**), and 12 h UV light/12 h dark cycles (**4**). (**5**) Sporodochia on SNA in vivo. (**6**,**7**) Sporodochial conidiophores. (**8**–**14**) Lateral monophialides. (**15**) Terminal chlamydospore. (**16**–**18**) Intercalary chlamydospores in hyphae. (**19**) Chlamydospores in conidia. (**20**) Conidia in air mycelium. (**21**) Conidia in sporodochia. (**22**) Typical range of conidia. Where not specified, scale is equal to 20 µm.

The radial growth rate is 6.1–6.5 mm/day. All the strains had floccose to felt-like, dense aerial mycelia that were white to yellow–white in color (Figure 3). In some cultures, small condensations of white or light-yellow sterile mycelium formed. The pigment is creamy and light yellow, but spots may appear on the reverse in the form of reflections of orange sporodochia on the surface of the medium. Sporodochial formation increases under light and UV light.

Lateral monophialides arise on aerial hyphae, are cylindric and subcylindric or doliform in shape and often have a collarette, (*n* = 58) 13.9 × 2.3 (5.7–34.4 × 1.7–3.5) µm.

Conidia are rather abundant in the aerial mycelium and form on single lateral monophialides. They do not aggregate into false heads, vary in shape (ovoid, fusiform, or crescent-shaped), and vary considerably in size, with 1–3 septa. Typically, conidia have pointed apical cells, and basal cells are distinctly pedicellate or have a small papilla. Additionally, some conidia terminate in distinctly swollen or onion-shaped tips. Aerial conidia size: 0 sept. (*n* = 36) 9.2 × 2.8 (6.4–13.7 × 1.9–3.6); 1 sept. (*n* = 36) 13.8 × 2.8 (10.0–19.4 × 2.3–3.4); 2 sept. (*n* = 2) 12.4–19.8 × 2.6–2.7; 3 sept. (*n* = 17) 17.6 × 3.3 (14.1–25.8 × 2.7–3.9) µm.

Later, the phialides rapidly branched irregularly or whorled and were tightly grouped, leading to the formation of sporodochia. Macroconidia from sporodochia are uniform, falcate, ventrally almost straight, and dorsiventrally slightly curved in the middle, narrowing to the basal cell, 3 (–7) septate. The apical cells of macroconidia are pointed, and basal cells are typically pedicellate or have papillae. Macroconidia size: 3 sept. (*n* = 58) 32.1 × 3.7 (18.5–40.5 × 2.9–4.7); 4 sept. (*n* = 17) 39.6 × 4.0 (33.0–45.1 × 3.2–4.6); 5 sept. (*n* = 1) 64.2 × 5.2; 6 sept. (*n* = 3) 46.2 × 4.5 (45.5–47.5 × 4.3–4.9); 7 sept. (*n* = 1) 45.5 × 4.6 µm.

Chlamydospores formed in hyphae and rarely in conidia and were mostly intercalary, being yellow or pale ochre, smooth- or rough-walled, solitary or in pairs, in chains, and typically unicellular; however, there are two-celled chlamydospores; (*n* = 52) 11.89 × 11.84 (7.3–17.5 × 5.8–18.0) µm in diameter. Sclerotia and an odor are absent.

***Fusarium* sp. MFG 13060**

**Figure 4:**
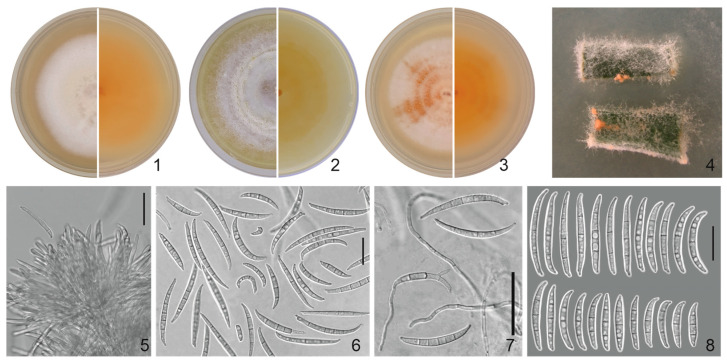
*Fusarium* sp. MFG 13060 on PDA at 25 °C in 1 week, dark (**1**); 3 weeks, dark (**2**); 1 week, 12 h UV light/12 h dark cycles (**3**). (**4**) Sporodochia on CLA. (**5**) Sporodochial conidiophores. (**6**–**8**) Typical range of conidia. Scale bar is 20 µm.

The radial growth rate is 4.7 ± 0.1 mm/day. The aerial mycelium is felt-like, dense, and white to cream. The pigmentation of colony varies in hue and chroma of yellow, with lightening toward the edge (Figure 4).

There are no typical microconidia, but few aerial conidia (1–2 septa) are observed in the mycelium. Sporulation occurs in the aerial mycelium, typically in aggregated sporodochia of varying sizes. They are initially light-cream-colored and become bright orange with age. Sporodochia formation increases under light and UV light.

In the CLA, bright-orange sporodochia are also abundant on densely aggregated, vertically branched conidiophores. Phialides are monophialidic, cylindric, and rather slender at 6.7 × 1.6 (2.2–25.4 × 1.5–3.0) µm.

From the sporodochia, macroconidia are uniform, falcate, and generally rather straight, with some slightly curved dorsiventrally and ventrally, and they have (1–2) 3–4 (–5) septa. The apical cells are elongated and slightly curved, and the basal cells are distinctly pedicellate or notched. Among the macroconidia, there are some with slightly enlarged basal cells.

Macroconidia measurements: 1 sept. (*n* = 3) 13.5 × 2.8 (11.7–16.6 × 2.6–3.0); 2 sept. (*n* = 2) 26.5 × 3.6 (14.8–38.2 × 3.5–3.6); 3 sept. (*n* = 56) 38.5 × 3.3 (22.4–51.4 × 2.7–4.1); 4 sept. (*n* = 23) 43.6 × 3.5 (33.6–53.1 × 2.9–3.9); 5 sept. (*n* = 7) 48.5 × 3.6 (43.9–59.3 × 3.2–4.1) µm.

There are no chlamydospores. Sclerotia and an odor are absent.

### 3.3. Mating Types and Crossing

Mating-type idiomorphs (*MAT1-1*/*MAT1-2*), which encode regulators of mating and sexual development, were identified for all the strains on the basis of the results of the PCR assay. The *F. heterosporum* strain MFG 58943 and *Fusarium* sp. MFG 13060 have the *MAT1-2* idiomorph. The *MAT1-1* idiomorph was found in the other three analyzed *F. heterosporum* strains.

**Figure 5:**
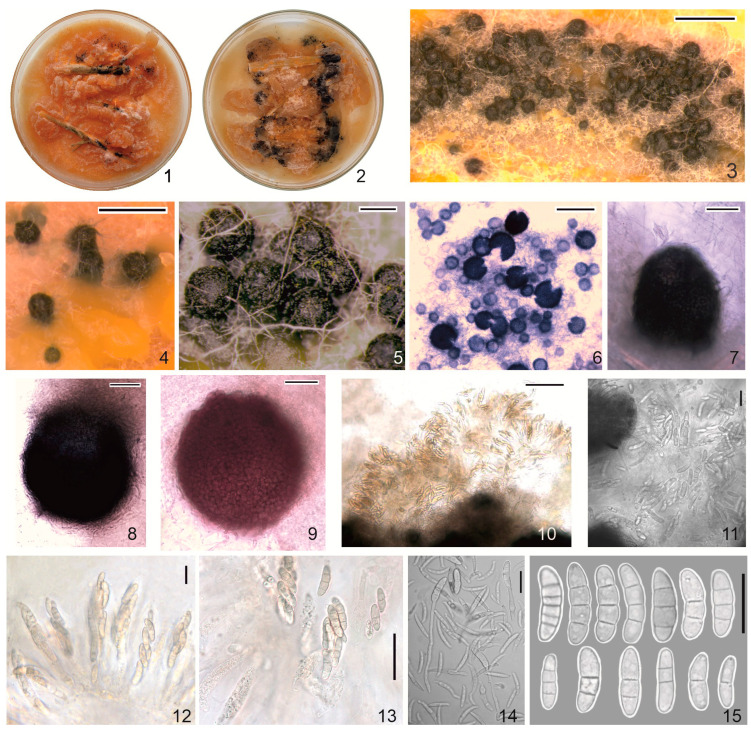
*Fusarium heterosporum* in crossing test: (**1**) MFG 58281 × MFG 58943 (2 weeks). (**2**) MFG 58248 × MFG 58943 (3 weeks). (**3**–**5**) Numerous perithecia on CMA (3 weeks). (**6**) Perithecia of different ages in drop of water. (**7**) Mature perithecium that releases asci through ostiole. (**8**) Perithecium. (**9**) Perithecium that changed color in lactic acid. (**10**–**13**) Asci with ascospores released from perithecia triggered by humidity. (**14**) Germinating ascospores in drop of water among macroconidia. (**15**) Ascospores. Scale bars: 3 = 1 mm; 4 = 500 µm; 5, 6 = 200 µm; 7, 10 = 100 µm; 8, 9 = 50 µm; all others = 20 μm.

In the results of coculture on CMA combinations of *F. heterosporum* strains of opposite mating types, MFG 58943 (*MAT1-2*) and the strains MFG 58273, MFG 58278, and MFG 58281 (*MAT1-1*), abundant mature perithecia were detected in two weeks on *Melilotus* stems and on agar close to them (Figure 5). Numerous bright-orange sporodochia of the fungus also formed on the surface of the CMA medium and on the pieces of the stem, covering them with a layer.

Perithecia were superficial, ovoidal to subglobose, solitary, later aggregated in the groups, and seated on a minute stromatic base. The perithecia were dark purple and blue–black, and the walls were angular and became red in lactic acid. The average size of the mature perithecia (*n* = 27) was 137.6 × 125.9 (100.4–337.1 × 99.6–276.9) µm.

When the perithecia were placed in a drop of water, asci with eight slightly curved ascospores quickly emerged from them. Asci are clavate and thin-walled, with inconspicuous apical rings, and have eight spores, measuring (*n* = 15) 123.8 × 17.5 (113.9–136.8 × 14.8–20.1) µm.

Ascospores are numerous, hyaline, slightly yellowish-brown when gathered in a mass, elliptical to fusoid and constricted at the septum, typically with three septa. However, ascospores with 1–2 and 5 septa could also be found, and they arose in a single ascus together with 3-septate ascospores.

Ascospore size: 1 sept. (*n* = 4) 13.6 × 3.9 (10.2–18.3 × 3.0–4.8); 2 sept. (*n* = 3) 22.5 × 6.1 (22.4–22.6 × 6.0–6.2); 3 sept. (*n* = 35) 22.0 × 5.6 (13.5–29.7 × 3.0–7.4); 5 sept. (*n* = 2) 20.3–26.4 × 5.2–7.7 µm.

The strain *Fusarium* sp. MFG 13060 carried one mating-type idiomorph (*MAT1-2*), and it did not mate with the analyzed *F. heterosporum* strains carrying *MAT1-1* under the tested conditions.

### 3.4. Effect of Temperature on Fungal Growth

**Figure 6:**
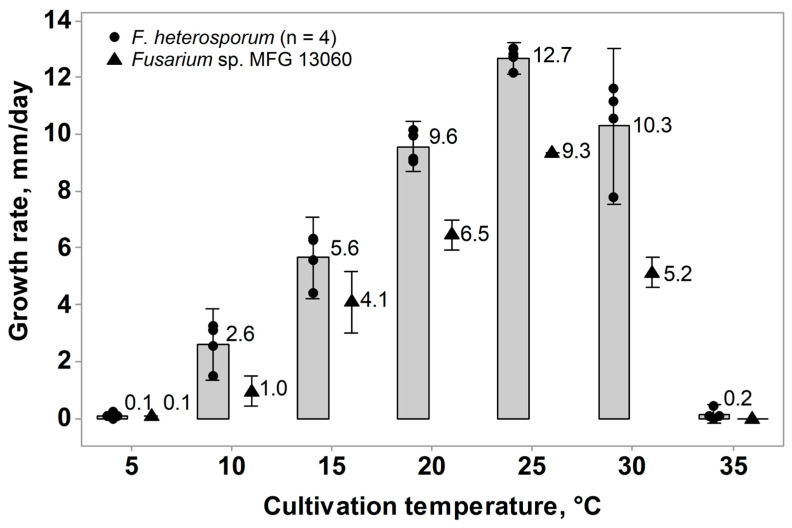
Growth rate of *Fusarium* strains belonging to FHSC at different temperatures (PDA, in dark). Bars with intervals are average value with confidence interval at significance level of *p* ˂ 0.05 for *F. heterosporum* strains; symbols mean values for individual strains.

At a temperature of 5 °C, the *F. heterosporum* strains, with the exception of MFG 58278, were able to grow on PDA (Figure 6), but the growth rate of the strains was relatively low (0.1–0.2 mm/day). In the temperature range of 10–30 °C, the *F. heterosporum* strains grew 1.4–2.7 times more actively than the strain *Fusarium* sp. MFG 13060. The optimal temperature for the growth of all the analyzed strains was 25 °C; when the diameter of the *F. heterosporum* colonies ranged from 73.0 to 78.0 mm, the diameter of the *Fusarium* sp. MFG 13060 colony reached 56.0 mm. When the temperature increased to 30 °C, a significant difference in the growth rate of *F. heterosporum* strains was detected: one strain, MFG 58943, grew more slowly (7.8 mm/day) than the other three strains of this species (10.5–11.6 mm/day). At a temperature of 35 °C, the growth of strains MFG 58273 and MFG 13060 stopped, whereas the other strains continued to grow slowly at a rate of 0.5–2.3 mm/day.

### 3.5. Toxin Production Ability of Strains

All five strains were examined for their ability to produce different mycotoxins typically synthesized by *Fusarium* fungi on an autoclaved rice substrate. No detectable levels of any of the analyzed mycotoxins were detected in the initial rice used for analysis. All the strains were able to produce only MON. The range of mycotoxin concentrations was 301.2–9688.9 μg/kg for *F. heterosporum* strains (on average, 4785.5 ± 2063.5 μg/kg), while *Fusarium* sp. MFG 13060 produced 15.1 μg/kg of MON.

## 4. Discussion

In this study, the taxonomic status of *Fusarium* strains previously identified as representatives of the *F. heterosporum* species complex on the basis of morphological features was confirmed using a phylogenetic analysis of sequences of two informative DNA loci for this group of fungi. The analyzed strains of *F. heterosporum* were isolated from ergot sclerotia on the panicles of wild grasses collected in two regions of Russia under different climatic conditions.

We confidently assumed that the MFG 13060 strain isolated from the stem of the dicotyledonous plant *Cirsium arvense* collected in the North Caucasian region belongs to the *F. graminum* species [3]. However, on the basis of molecular genetic data, this strain, together with the strain BBA 62226 isolated from *Claviceps* sp. on the ear of *Paspalum dilatatum* in Iran and previously morphologically identified as *F. graminum* [29], formed a distinct phylogenetic lineage. In the future, with the increasing number of similar strains and more detailed studies of their characteristics, it will become possible to describe new taxa within the FHSC.

The species epithet of *F. heterosporum* is entirely consistent with the existing diversity of morphological structures in fungal strains of this species, including conidia, ascospores and chlamydospores. Although we did not identify typical microconidia in the strains, a fairly large number of conidia of various sizes and rather variable forms quickly developed in the aerial mycelium. Additionally, the rapid formation of sporodochia with 3–4-septate macroconidia was observed. In the strain *Fusarium* sp. MFG 13060, conidia were nearly absent in the aerial mycelium, but mostly 3–4-septate macroconidia also formed quite quickly in the sporodochia. Thus, these two *Fusarium* species, which are outwardly inconspicuous, lacking abundant aerial mycelia and weakly colored in soft yellow shades, are characterized by the rapid formation of brightly colored sporodochia.

With growth on SNA for 2 weeks, 54% of macroconidia had three septa of the strain *Fusarium* sp. MFG 13060, whereas those of the *F. heterosporum* strains consisted of predominantly unicellular conidia and also one- and three-septa conidia. The length-to-width ratio of three-septate macroconidia in *F. heterosporum* strains was 8.7, whereas that of four-septate macroconidia was 9.8. In strain MFG 13060, this ratio was also 11.6 and 12.3 for three- and four-septate macroconidia, respectively.

In the strains of *F. heterosporum* on average, the chlamydospores were quite large, but there was a noticeable difference in size and shape; the difference between large and small chlamydospores ranged from 2.4 to 3.1 times. Furthermore, the presence of two-celled chlamydospores is not typical of *Fusarium* species. We did not observe chlamydospores in the studied strain MFG 13060. While the absence of chlamydospores is indeed a key characteristic in *Fusarium* fungi, their formation can vary depending on the growth conditions or age of the culture [8,25].

The plant tissue of *Melilotus album* effectively stimulated the formation of the sexual stage of *F. heterosporum*. It has been previously shown that *Fusarium* [*Gibberella*] species readily form perithecia in artificial culture on various media, such as carrot agar [8], twigs of *Morus alba* (mulberry) [30], and wheat straw [6,7].

The crossing of opposite MAT types and abundant production of fertile perithecia clearly revealed that *F. heterosporum* is heterothallic (self-sterile) and that the analyzed strains have different functional alleles of the MAT locus. Slightly curved and mainly three-septate ascospores are produced by *F. heterosporum*, which is typical for *F. graminearum* and most other species of the *F. sambucinum* species complex for which sexual stages are noted, in contrast to the one-septated ascospores found in the *F. fujikuroi* species complex [18]. Moreover, the strain MFG 13060, which carries one mating-type idiomorph, did not mate with the *F. heterosporum* strains used in this study under the tested conditions.

A teleomorph stage for *F. heterosporum* was first reported by the Canadian mycologist W.L. Gordon in 1961. He called it *Gibberella cyanea* (Sollm.) Wollenw., and later, C. Booth redescribed it as *G. gordonia* [6]. The perithecia obtained in our study were smaller at the lower border than those reported [6], the asci were similar in size, and the ascospores were significantly longer and wider. One possible reason for such large ascospores could be their rapid swelling in a drop of water. In a short period of the visualization of the morphological structures under the microscope, germinated ascospores were already found, each with a single hypha. It is likely that such ascospores will be larger than ungerminated ones.

To the best of our knowledge, only two studies in which strains of this species were crossed exist. First, W.L. Gordon described the *Gibberella* perithecial state of *F. heterosporum* and reported that it was hermaphroditic but self-sterile [31]. However, owing to the complexity of morphological identification and, as a consequence, the blurred boundaries of the species, there is no certainty in the correct identification of these strains. Later, Australian researchers, who considered *F. graminum* and *F. heterosporum* to be synonymous taxa, suggested, albeit with some doubts, the possible homothallism of these fungi on the basis of the results of crossbreeding experiments [7]. The obtained perithecia were characterized as barren, since only occasional ascospores, frequently appearing as misshapen, were found.

Notably, the entire life cycle of the fungus has been achieved in culture, but it has not been found in nature. Interestingly, the *F. heterosporum* strain from the northwestern region of Russia carried the *MAT1-2* idiomorph, whereas in three strains from the southern region of Russia, the MAT locus was represented exclusively by the *MAT1-1* idiomorph. Of course, a limited number of strains have been studied, but it is possible that certain MAT idiomorphs are distributed across geographically distant regions.

According to our data, the strains can grow across a wide range of temperatures (10–30 °C). *Fusarium heterosporum* appears to be ubiquitous on cereal plants, but it is a relatively little-studied fungal species. This may be due not to its rarity in the mycobiota of plants but to its rare identification by researchers.

The habitat specialization of *F. heterosporum* to plants, if any, is mostly unknown. To date, there is no clear understanding of the host range for this fungus: it is predominantly found on cereal plants; sometimes in publications and collections, there are references to this taxon for various plants, but this information needs confirmation. Since *F. heterosporum* has been shown to be associated with ergot sclerotia, it can be assumed that there is a possible ecological association between this fungus, *Claviceps* spp. and cereal plants. *Claviceps* fungi are ubiquitous pathogens of monocotyledonous plants, in which the grains are replaced by fungal sclerotia containing alkaloids that can cause severe poisoning in mammals [32]. *Fusarium heterosporum* was found in association with the ergots on *Spartina anglica*, and the authors suggested that the negative effect of *Fusarium* on *Claviceps* spp. was insignificant [14].

Problems with the identification and toxigenic potential of *F. heterosporum* have been repeatedly documented. The first detection of fusaric acid, a major toxic metabolite of *Fusarium* fungi, was associated with efforts by the Japanese chemist T. Yabuta, who in 1934 obtained a crystalline substance from the filtrate of a *F. heterosporum* that was later re-identified as *Gibberella fujikuroi* [33]. Later, screening revealed that the ability of *Fusarium* strains of different species to produce this mycotoxin was the greatest in the *F. heterosporum* strain isolated from Bermuda grass (*Cynodon dactylon*).

According to Cole et al. [34], the isolate of *F. heterosporum* parasitizing honeydew and immature sclerotia of *Claviceps paspali* was highly toxic to chickens, and the presence of six trichothecenes, including T-2 toxin, HT-2 toxin, and T-2 triol, was detected by spectroscopic methods. However, subsequent studies of the two strains of *F. heterosporum* and *F. graminum* failed to show chemical or genetic evidence of the ability of these fungi to produce major *Fusarium* mycotoxins [35]. A single report on the ability of one *F. heterosporum* strain to produce the specific mycotoxin equisetin was also published [36].

It is common practice to use autoclaved rice grains and crushed maize kernels, as well as PDA, to analyze the ability of *Fusarium* strains to produce mycotoxins; however, grain substrates are noted as the most favorable for more abundant production and diversity of metabolites synthesized by fungi [37]. In this study, we cultivated the strains on autoclaved rice, and under these experimental conditions, only MON was detected out of 19 analyzed mycotoxins in all the *F. heterosporum* and *Fusarium* sp. MFG 13060 strains. Previously, MON was not detected in the mycotoxin profiles of five *F. heterosporum* strains [38].

Moniliformin is a common contaminant in cereals and cereal-based products and is produced by the fungi of the *Fusarium tricinctum* species complex (*F. avenaceum*, *F. acuminatum*, *F. tricinctum,* etc.) and the *Fusarium fujikuroi* species complex (*F. proliferatum*, *F. verticillioides*, *F. subglutinans, F. thapsinum,* etc.) [39,40,41,42]. The hematotoxicity and cardiotoxicity of MON was reported in a potential risk assessment [43]. Furthermore, the possible additive or synergistic toxic effects of MON and ergot alkaloids on the health of consumers when they are simultaneously present in plant material cannot be excluded.

The production of secondary metabolites in *Fusarium* fungi is highly variable and depends on many abiotic and biotic factors. The significant effects of fungal species, strain, host, substrate, and environmental conditions (temperature, water activity, etc.) on MON production by fungi have been repeatedly demonstrated [40,44,45,46,47]. Currently, owing to the identification of strains using molecular genetics, it is possible to clarify the specific characteristics of species, including the precise determination of the spectrum of their toxic metabolites.

## 5. Conclusions

This study attempted to clarify the species boundaries of the *F. heterosporum* taxon, including data from phylogenetic and morphological analyses, ecological characteristics, and mycotoxin production. The four strains isolated from cereal plants with signs of ergot from two different regions of Russia were identified as *F. heterosporum*. One genetically closely related *Fusarium* sp. within the *Fusarium heterosporum* species complex was isolated from *Cirsium arvense*. Heterothallism and the ability to undergo sexual reproduction in the species *F. heterosporum* were confirmed. When all the strains were cultivated on autoclaved rice, their ability to produce only moniliformin out of 19 analyzed *Fusarium* mycotoxins was established. Considering modern identification technologies, further research is needed to elucidate the species diversity and geographic distribution of the representatives in the FHSC and to clarify the unclear relationships between *Fusarium* and *Claviceps* fungi.

## Figures and Tables

**Table 1:** Information on *Fusarium* strains analyzed in this study.

Species	Strain Number ^1^	Origin: Region, Location ^2^	Substrate, LEP Number ^3^	Year	GenBank Accession Number	
					*tef*	*rpb2*
*Fusarium* sp.	MFG 13060	North Caucasian, Stavropol Krai	*Cirsium arvense*, ―	2006	PQ459027	PQ459046
*F. heterosporum*	MFG 58273	North Caucasian, North Ossetia	*Cynodon dactylon*, LEP 65303	2010	PX353839	PX353842
*F. heterosporum*	MFG 58278	North Caucasian, North Ossetia	*Poa* sp., LEP 65304	2010	PX353840	PX353843
*F. heterosporum*	MFG 58281	North Caucasian, North Ossetia	*Elytrigia* sp., LEP 65301	2010	PX353841	PX353844
*F. heterosporum*	MFG 58943	North Western, Leningrad Oblast	*Lolium* sp., LEP 65302	2016	PQ459028	PQ459047

^1^ Strain number in the collection of the Laboratory of Mycology and Phytopathology of All-Russian Institute of Plant Protection (St. Petersburg, Russia). ^2^ Region means the Federal District of Russian Federation. ^3^ Specimen number in the Mycological Herbarium LEP. A dash indicates the absence of LEP number.

**Table 2:** The reference FHSC strains included in the phylogenetic study.

Species	Strain Number ^1^	Origin	Substrate	GenBank Accession Number	
				*tef*	*rpb2*
*F. graminum*	CBS 737.79 = BBA 62228 = DSM 62228 = IMB 11721 = MRC 1792 = NRRL 20692	Ethiopia	*Cynodon dactylon*, ear	JAAGWP010000622	JX171593
*F. graminum*	BBA 62226	Iran	*Claviceps* sp. on ear of *Paspalum dilatatum*		HQ728166
*F. heterosporum*	ASV994	Italy	wheat grain	PV054603	
*F. heterosporum*	CBS 391.68 = NRRL 25798 T ^2^	Germany	*Claviceps purpurea* sclerotium on *Lolium perenne*	MW928839	MW928827
*F. heterosporum*	CBS 720.79	Netherlands	*Claviceps purpurea* sclerotium on *Lolium perenne*	JAAGWQ010000739	JX171594
*F. heterosporum*	DAOMC 235644			KR909339	
*F. heterosporum*	ICMP 11686 = NRRL 39891	New Zealand	*Claviceps* sp.	MG857401	
*F. heterosporum*	ICMP 11865	New Zealand	*Ulex europaeus*	MG857464	
*F. heterosporum*	ICMP 11866	New Zealand	*Ulex europaeus*	MG857465	
*F. heterosporum*	ICMP 4694 = NRRL 39574	New Zealand	*Lolium* sp.	MG857202	
*F. heterosporum*	ICMP 4907 = NRRL 39547	New Zealand	*Zea mays*	MG857186	
*F. heterosporum*	ICMP 5156 = NRRL 39542	New Zealand	*Paspalum dilatatum*	MG857183	
*F. heterosporum*	ICMP 5427 = NRRL 39652	New Zealand	*Paspalum* sp.	MG857256	
*F. heterosporum*	ICMP 5655 = NRRL 39692	New Zealand	*Lolium perenne*	MG857282	
*F. heterosporum*	ICMP 10542	New Zealand	*Lolium*	EU327335	
*F. heterosporum*	ICMP 10543 = NRRL 39835	New Zealand	*Solanum lycopersicum*	MG857363	
*F. heterosporum*	PDD63	New Zealand	*Triticum aestivum*	EU327338	
*F. nurragi*	CBS 393.96 = DAR 69502 = NRRL 36453	Australia	soil	MW928840	MW928830
*F. qiannanense*	GUCC 198067.1 = CGMCC 3.25477 T	China	healthy roots of *Rosa roxburghii*	OR043905	OR043849
*F. qiannanense*	GUCC 198067.2	China	healthy roots of *Rosa roxburghii*	OR043906	OR043850
*F. qiannanense*	KUNCC 3415	China	*Claviceps purpurea* sclerotium	PV387321	PV464077
*F. qiannanense*	KUNCC 3416	China	*Claviceps purpurea* sclerotium	PV387323	PV464078
*F. qiannanense*	KUNCC 3417	China	*Claviceps purpurea* sclerotium	PV387322	PV464079

^1^ Strain number in the collection: BBA—Biologische Bundesanstalt für Land- und Forstwirtschaft, Braunschweig, Germany; CBS—Culture collection of the Westerdijk Fungal Biodiversity Institute, Utrecht, The Netherlands; CGMCC—Chinese General Microbiological Culture Collection Center, Beijing, China; DAOMC—Canadian Collection of Fungal Cultures, Ottawa, Canada; DSM—Deutsche Sammlung von Mikrorrganismen und Zellkulturen GmbH, Braunschweig, Germany; GUCC—Culture Collection at the Department of Plant Pathology, Agriculture College, Guizhou, China; ICMP—International Collection of Microorganisms from Plants, Auckland, New Zealand; IMB—Institut für Mykologie, Berlin-Dahlem, Germany; KUNCC—Kunming Institute of Botany Culture Collection, Kunming, China; MRC—PROMEC, Medical Research Council, Tygerberg, South Africa; NRRL—Agricultural Research Service Culture Collection, National Center for Agricultural Utilization Research, Peoria, USA; PDD—Plant Disease Division herbarium, New Zealand Department of Scientific and Industrial Research, Aukland, New Zealand; ^2^ T—type strain.

## Data Availability

All data is contained within the article.
